# Effects of covid-induced lockdown on inhabitants’ perception of indoor air quality in naturally ventilated homes

**DOI:** 10.1007/s11869-022-01239-3

**Published:** 2022-10-03

**Authors:** Jesica Fernández-Agüera, Samuel Domínguez-Amarillo, Miguel Ángel Campano, Hanan Al-Khatri

**Affiliations:** 1 Escuela Técnica Superior de Arquitectura, Instituto Universitario de Arquitectura y Ciencias de La Construcción, Universidad de Sevilla, Avenida de Reina Mercedes, no. 2, 41014 Seville, Spain; 2 Department of Civil & Architectural Engineering, College of Engineering, Sultan Qaboos University, Seeb, Oman

**Keywords:** Airtightness, Indoor air quality, Occupants’ perception, Low-income housing, Ventilation behaviour, Health effects, COVID-19, Lockdown

## Abstract

The intensified indoor living during the spring 2020 lockdown, with enhanced user awareness of the prevailing conditions in their homes, constituted a natural stress test for the housing design in place today. Surveys conducted during this period have yielded lessons for designing better intervention strategies for the residential sector, taking into account the systematic morphological and economic limitations of the buildings concerned. These considerations should inform the development of policies and strategies for improving environmental quality compatible with lower residential energy consumption and higher quality of life. This study explores the effect of occupant behaviour on home ventilation and the perception of the impact of indoor air quality on user health before and during lockdown. The method deployed consisted in monitoring environmental variables and conducting user surveys before and after restrictions came into force. The findings showed that prior to lockdown, occupants were unaware of or paid little heed to changes in indoor air quality, failed to perceive stuffiness, and, as a rule, reported symptoms or discomfort only at night during the summer months. During lockdown, however, users came to attach greater importance to air quality, and a greater sensitivity to odours and a heightened awareness of CO_2_ concentration prompted them to ventilate their homes more frequently. In the spring of 2020, occupants also indicated a wider spectrum of indisposition, in particular in connection with sleep patterns.

## Introduction

Recent research has shown that outdoor air pollution causes around 3 million premature deaths yearly worldwide (Organization 2014). It also affects indoor air quality, particularly in cities with high pollution levels (Linares et al. 2009; Reiminger et al. 2020). The APHEKOM Project reported the exposure of Europeans to pollutant concentrations in excess of World Health Organization (WHO) recommendations and put forward estimates of the potential economic benefits of the reduction in urban air pollution in Europe (Pascal et al. 2013). 33% of citizens in large Spanish cities such as Seville and Madrid are exposed to pollution higher than the values provided in Directive 2008/50/EC and Spanish Royal Decree 102/2011, while 95% breathe air with levels above the thresholds recommended by the WHO (Ecologistas en accion 2021).

In recent years, these developments have brought indoor air quality (IAQ) in homes to the attention of the general public (Angell et al. 2005), bearing in mind that the perception of IAQ can be affected by the country of origin of the occupants (Humphreys et al. 2002), as well as by temperature, humidity, and air velocity (Alwetaishi and A Balabel 2016; Humphreys et al. 2002; Nicol and Humphreys 2002; Roelofsen 2018). A number of studies have been conducted in the UK on the relationship between IAQ (Abdalla and Peng 2021) and ventilation systems (McGill et al. 2015, 2016; Sharpe et al. 2016; Sherman et al. 2012), while the impact of deep energy retrofits (DERs) on IAQ has been analysed in Ohio, USA (Wells et al. 2015). French researchers published a study on timber homes entitled ‘Indoor air quality in energy-efficient dwellings: levels and sources of pollutants’ (Derbez et al. 2018), as well as an evaluation of the perceived IAQ in dwellings related to measurements of gaseous and particulate matters (Langer et al. 2017), research also carried out in Silesia (Poland) evaluating the risk perception related to exposure to indoor environmental factors for 3 months with 552 subjects (Krupa et al. 2012). In addition, Chinese researchers have linked Parents’ Perceived IAQ in dwellings to childhood diseases such as allergies (Qian et al. 2016). Different items of internal equipment used for cooking such as natural gas cooking burners (Amirkhani Ardeh et al. 2020) or cooking stoves (Amouei Torkmahalleh et al. 2017) greatly influence indoor air pollution and can cause adverse health effects. Furthermore, the influence on IAQ of the design of open-plan cooking in tiny substandard homes has also been highlighted (Cheung et al. 2019).

In a broader context, using an integrated approach to indoor environmental quality (IEQ), some studies in more temperate climates also establish a correlation of the interactions between lighting — especially the correlated colour temperature (CCT) and the lighting level thought the hue-heat hypothesis — and thermal comfort (Bellia et al. 2021; te Kulve et al. 2018) which also can affect user productivity (al Horr et al. 2016; Bueno et al. 2021; Rasheed and Byrd 2017; Wargocki and Seppänen 2006), parameter that is becoming increasingly important due to the rise of teleworking, a fact that has been accelerated due to the health situation of COVID-19. However, these issues have been largely neglected under the assumption that windows in homes are open most of the year, mainly exposed to natural lighting. In these circumstances, user behaviour in housing is of considerable importance and highly dependent upon outdoor weather conditions.

In fact, during the COVID-19 pandemic, indoor air quality has come to the fore due to the airborne transmission of SARS-CoV-2 (Greenhalgh et al. 2021; Kutter et al. (n.d.), as well as to the impact of the lockdown on the reduction of air pollution and particle matter levels at the residential (Jakovljević et al. 2021) and urban measurements (Girdhar et al. 2021; Teixidó et al. 2021). This virus spreads predominantly through aerosols, mainly in indoor environments, as analysed in several studies (Kohanski et al. 2020; Prather et al. 2020a, b; Prather et al. 2020a, b; Qian et al. 2021; Tang et al. 2021). Therefore, the greater the number of people and time of permanence in the same space, the greater the statistical possibility of contagion. Therefore, the probability of infection increases by up to 30% among close household members, (López et al. 2021). Although close contact between cohabitants is the main cause of contagion, this may also occur between different dwellings, especially through vertical building drainage stacks. Contagion events have been documented by both the chimney effect-induced airflow in building drainage systems, especially in high-rise housing (Wang et al. 2022), and through faecal aerosol transmission from toilets and washbasins (Kang et al. 2020). This is why some studies have analysed the impact of the isolation of COVID-19 patients in community-supervised facilities, which may protect their household members from transmission of the disease (López et al. 2021). Despite these, contagion of susceptible occupants from hotel-isolated patients can still occur through shared air from corridors, HVAC systems, and drainage stacks (Gu et al. 2022; Hoefer et al. 2020; Leong et al. 2021).

An indirect relationship can be established between the risk of airborne contagion and poor air quality. Thus, since CO_2_ is co-exhaled with aerosols (which can contain virions from infected occupants), it can be used as a proxy to estimate statistically the risk of airborne infection indoors. The Wells-Riley transmission model (Rudnick and Milton 2003) analyses the risk of transmission of multiple airborne diseases through the number of ‘quanta’ (amount of infectious doses of the virus required to cause infection in 63% of susceptible people) inhaled by a susceptible person during the event under analysis. This method, adapted to SARS-CoV-2 and successfully validated by comparing COVID-19 super spreading events (Peng and Jimenez 2021), is used to develop a calculation system for the evaluation of other hypothetical events (Campano-Laborda et al. 2021).

However, the level of CO_2_ can have a greater impact on occupants’ health, not just as a mere indirect indicator of environmental quality or disease transmission. Staying in a room with moderate-to-high CO_2_ concentrations may also affect cognitive processes and decision-making performance, and a degradation of multiple cognitive measures can be detected when CO_2_ values exceed 1000 ppm (Allen et al. 2016; Satish et al. 2012).

This study aims to assess the performance of the building envelope for a healthy environment. The behaviour of the occupants and variations in indoor air quality have been analysed under normal conditions and in periods of high intensity of use. This analysis considers the evaluation of the permeability performance of the building envelopes, its relationship to indoor air quality conditions, and its influence on occupants’ perception and health before and during lockdown.

These residential buildings dating from 1940–1979 have envelopes characterized by fairly low energy performance. The façade and roof enclosures normally lack thermal insulation, and a significant percentage of the façades consist of single-wythe masonry with no air cavities. The windows generally have very simple joinery and non-insulating glazing, except where recently replaced (Domínguez-Amarillo et al. 2016; Ignacio Oteiza; Carmen Alonso; Fernando Martín-consuegra 2019). The flats in these buildings depend on natural ventilation (open windows) and the uncontrolled supply of outdoor air attributable to infiltration (Fernández-Agüera et al. 2016, 2019; Feijó-Muñoz et al. 2018). Therefore, maintaining acceptable indoor conditions requires considerable energy consumption, which is particularly costly for low-income users. In light of the need to provide these families with comfortable conditions at an affordable cost, energy rehabilitation is a matter of socio-economic as well as environmental importance.

In the residential sector, reducing energy consumption for environmental control by up to 50% (European Commission 2011) calls for considerable technical know-how, given the wide range of circumstances to be addressed. Efforts will focus on enhancing façade performance to reduce demand. The rehabilitation works envisaged or underway to increase airtightness in envelopes (d’Ambrosio Alfano et al. 2016; Pampuri et al. 2018; Suárez and Fernández-Agüera 2011) fail to make allowances for the ventilation required in dwellings with no mechanical ventilation affecting indoor air quality.

Increasing the airtightness of buildings may help to achieve indoor thermal comfort. However, based on the above, this is unfortunately associated with health concerns. Thus, it is crucial for increased building airtightness to maintain the levels of natural ventilation required. The data and analyses presented here will contribute to expanding the information on the effects and the perception of the occupants of the air quality of their homes, an aspect of particular interest, especially now that a global pandemic is forcing us to stay at home.

## Methodology

This study was part of a nationwide project implemented in two Spanish cities: Madrid and Seville. The sampling of dwellings and constructive characterization are described in detail in (Domínguez-Amarillo et al. (2016, 2017). Air temperature, relative humidity, and CO_2_ concentration were measured in the dwellings studied. In addition, for a full year and during the lockdown period, participants were asked to complete a questionnaire on ventilation habits, occupant comfort, sleep quality, and health symptoms. This study was approved by the relevant local ethics committees, and all participants provided informed consent.

### Location and climate

**Fig. 1:**
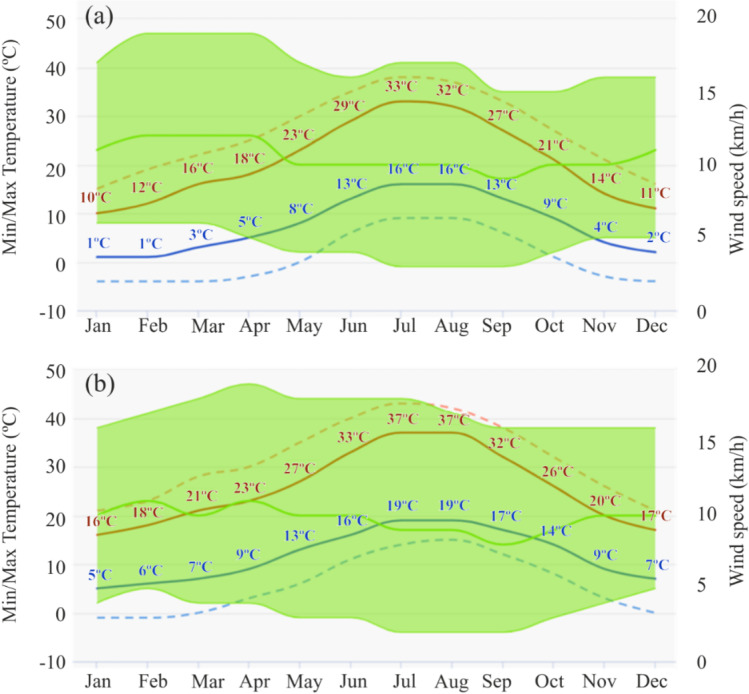
Air temperature and wind speed values in Madrid (**a**) and Seville (**b**), obtained from Spain’s National Meteorological Agency. Legend: Maximum mean daily air temperature (red); maximum daily air temperature (dashed); minimum mean daily air temperature (blue); minimum daily air temperature (dashed); and wind speed (green)

Seville has a Mediterranean climate, with warm summers and temperate winters, while the climate in Madrid is more continental, with colder winters and somewhat cooler summers (de la Flor et al., 2008). However, both cities are considered as Csa in the Köppen climate classification (Kottek et al. 2006). In more general terms, the warm season can be said to prevail in Seville, whereas the cold one is more prevalent in Madrid. These features must be borne in mind when characterizing user behaviour and the facilities that the dwellings are equipped with. The air temperature and wind speed values in Seville and Madrid can be seen in Fig. 1.

### Definition and identification of the target population

There are currently no comprehensive extensive global data on social housing stock, either at national or regional level. This lack of data has made it necessary to carry out prior identification and characterization work on the properties built from 1939 to 1979. Information and documentation compiled have been included in a database of the location and specific characteristics of the properties (morphology and construction, dimensions, components and other aspects) in the eight provinces of the Autonomous Region of Andalusia. These studies are explained in detail in (Alonso et al. 2018; Domínguez-Amarillo et al. 2017). The target population was defined and identified in order to select the most representative cases from the study samples.

### Sample definition

Based on the findings of these studies, three representative city neighbourhoods studied were sampled in both cities. The individual dwellings surveyed were chosen based on occupant agreement, as residents’ collaboration was necessary in the monitoring and survey process.

Table 1 shows the characteristics of the different dwellings and the occupational profiles of the study samples selected. As there is a wide range of occupational profiles, including couples without children and full families, the divergences in the patterns of use can be compared. Given that none of the dwellings had a ventilation system air renewal within them depends on window opening by occupants. Dwelling M2 underwent an energy retrofit, with insulation added to the envelope and windows replaced, paying special attention to possible infiltrations. However, in the case of S3 cladding elements in the interior of the house were improved without taking into account aspects relating to infiltrations and the inclusion of air conditioning systems.

**Table 1:** Household profiles

	M1	M2	M3	S1	S2	S3
City	Madrid	Madrid	Madrid	Seville	Seville	Seville
Year	1960	1973	1965	1963	1968	1964
Façade	1-ft brick	0.5-ft brick + air space + partition wall system	A 1-ft brick + air space + partition wall system	1-ft brick	0.5-ft brick + air space + partition wall system	A 1-ft brick + air space + partition wall system
No. Of occupants	3	4	4	3	2	4
User profile	3 adults	Couple with children	4 adults	Adults	2 adults	Couple with children
Retrofitting	No	Yes	No	No	No	Yes
Ventilation system	No	No	No	No	No	No
Heating system	Gas boiler	Gas boiler	Central heating	No	One split AC units	Electric radiators
Cooling system	No	No	No	No	Two split AC units	Dx splitducted unit

### Airtightness and ventilation evaluation

Two approaches were used to determine building envelope airtightness and ventilation rates, the blower door test, and the tracer gas method. The tracer gas in this case was the CO_2_ exhaled by occupants. This is an alternative to the traditional release of gases during testing and is a better measure of the changes in indoor air that are attributable to infiltration. The findings can also be used to establish the actual variation in uncontrolled ventilation owing to flat location and orientation, differences in indoor temperature, and the wind profile during measurement.

#### Blower door test

Airtightness tests were carried out with Blower Door equipment, following the indications of standard EN-ISO 9972:2015 (ISO 2015) and specific protocols (UNE-EN 13,829:2002 n.d.). Method A of this regulation was selected to evaluate the operational status of the dwelling. Since these are multi-family buildings, each dwelling was measured as an independent unit. According to studies in these homes, air infiltrations through adjacent dwellings are less than 5% of the total amount measured (Fernández-Agüera et al. 2016).

#### Tracer gases

As an alternative to pressurization tests, which provide data on airtightness in non-operating conditions in the home, tracer gas tests are used to determine ventilation speeds, measure infiltrations of the existing air in the building under normal work conditions, study air movement and dispersion of pollutants, and identify areas with stagnant air.

The basic principle of this system is to release a quantity of a gas, called tracer gas, into the air to replace it and study its behaviour. This makes it possible to obtain approximate representative conditions of the real situation in order to carry out different studies and measurements on ventilation. These tests take longer and require far more sensitive and expensive instruments which are difficult to operate.

The internationally used standard for carrying out these tests, ASTM E741 (ASTM International 2019), describes the various gas test methods with tracers based on concentration drop, emission, and constant concentration measurements. It also lists tracer gases that can be used, along with their toxicology, chemical reactivity, and ability to be detected. It provides details on the calibration of gas analysers and how to perform analyses to determine errors made when testing with tracer gases. However, in Spain, the good practice guide NTP 345 (Gracia et al., n.d.) provides a more concise description of the different tracer gas methods.

### Indoor air quality measurements

The indoor air quality (IAQ) inside the dwellings studied was monitored while these were occupied. This in turn made it possible to consider the influence of the inhabitants’ behaviour on the environmental parameters. It should also be noted that the measured IAQ parameters were used to calibrate the simulation models and to establish relations to permeability.

The IAQ measurements were carried out for 1 year in all case studies and recorded in the main bedroom and the living room using a Wohlër CDL 210 CO_2_ datalogger with an accuracy of ± 3% (RH), ± 0.6 °C (*t*_*a*_), ± 3% of reading, or ± 50 ppm of CO_2_. All indoor air quality measurements were carried out in accordance with ISO 16000. In addition, outdoor variables were obtained from Spain’s National Meteorological Agency. The period studied during lockdown extended from 15th March to 26th April 2020, when the stay-at-home order in place meant that people were only allowed to leave their homes in exceptional cases.

### Occupant behaviour and perception

Occupants were surveyed about the comfort felt and their perception of indoor air quality in summer, winter, and the intermediate seasons in the living room (daytime area) and bedroom (night-time area) as presented in the Appendix. There was one questionnaire for each household, one for each occupant (adults), and one for each child under 8 (to be completed by a parent). There were also questions about average occupancy in the living room/kitchen and in the home every hour in addition to hourly activities such as heating, cooking, use of cleaning products, cosmetics, opening doors/windows, use of domestic heating/cooling, and ventilation habits. In each city, the questionnaire was distributed twice over four different seasons, along with a psychological test developed by the Centre for Sociological Research (CIS), Survey on the mental health of Spanish people during the COVID-19 pandemic (CIS 2021).

### Limitations

The sample consisted of low-income housing occupied primarily by the elderly or young couples with small children with insufficient resources to ensure year-round comfort in their homes, which had been built in a period (1939–1979) when housing lacked both insulated envelopes and mechanical ventilation. The small sample size in both cities was in keeping with this initial attempt to implement a novel approach. The results obtained for occupant perception and ventilation rates during lockdown may be partially influenced by seasonal conditions (spring).

## Results

### Airtightness

**Fig. 2:**
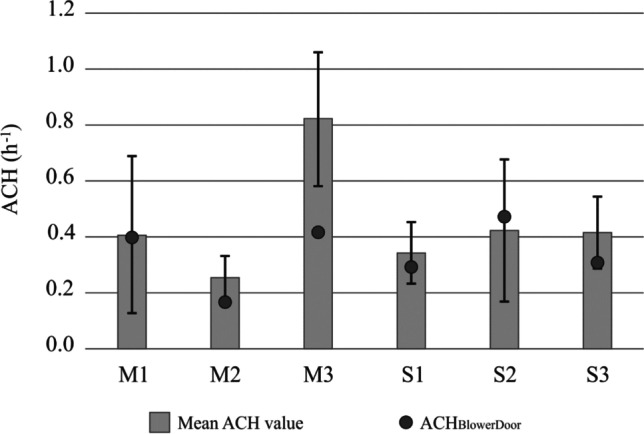
Flat airtightness data of dwellings in Madrid (M) and Seville (S): mean ACH values and ACH_BlowerDoor_

The air changes per hour in each dwelling is a very important parameter in winter, as air changes through the envelope are their main ventilation mechanism, along with opening windows, but only between 10 and 20 min daily as will be detailed later. The number of air changes through envelopes depends on temperature, wind velocity, and dwelling orientation and geometry. The ACH values for the winter period are set out in Table 2 and Fig. 2. Homes located in the same city exhibited different *n*_50_ values, with greater mean values than when the formula was used (Eq. 1). It is worth mentioning that *n*_50_ measures the number of air changes per hour through the building envelope at a reference pressure of 50 Pa.

**Table 2:** Flat airtightness data

	M1	M2	M3	S1	S2	S3
n_50_	7.90	3.20	8.30	5.69	11.02	6.10
ACH_BlowerDoor_	0.40	0.16	0.42	0.29	0.55	0.31
Mean ACH	0.40	0.35	0.82	0.34	0.42	0.41
Minimum ACH	0.13	0.15	0.39	0.15	0.20	0.36
Maximum ACH	0.91	0.43	1.14	0.57	0.90	0.79
Standard deviation	0.28	0.08	0.24	0.11	0.26	0.14

1 $${\mathrm{ACH}}_{\mathrm{BlowerDoor}}=\mathrm {n}_{50}/20$$

Table 2 shows the *n*_50_ values extracted from the Blower Door test carried out according to the protocol established by UNE-EN ISO 9972: 2019. The ACH_BlowerDoor_ values are the result of applying simplified formula 4 to the Blower Door tests performed. The maximum, minimum, and mean ACH values have been found using CO_2_ as a tracer gas. This CO_2_ has been measured continuously throughout the year, and this measure was established for closed dwellings without occupants.

It is observed that the mean ACH values measured with tracer gases are usually higher than those found directly following the application of the simplified formula in Madrid. However, in Seville, the lower values observed in the most air-permeable dwelling may be due to the wind profiles and the position or orientation of the dwelling.

### Occupant behaviour

The occupancy rates of the dwellings before and during lockdown are presented in Table 3. Before lockdown, the occupancy rate of the dwellings was 66–75% in Madrid, compared to 41–58% observed in Seville. During lockdown, the occupants stayed at home 100% of the time and many of them worked from home as they were not key workers.

**Table 3:** Occupancy rate of the dwellings before (BF) and during lockdown (LD)

Hour		1	2	3	4	5	6	7	8	9	10	11	12	13	14	15	16	17	18	19	20	21	22	23	24
M1	BF	3	3	3	3	3	3	3	2	2	1	1	1	1	0	0	2	2	2	2	2	3	3	3	3
	LD	3	3	3	3	3	3	3	3	3	3	3	3	3	3	3	3	3	3	3	3	3	3	3	3
M2	BF	4	4	4	4	4	4	4	4	0	0	0	0	0	0	1	1	3	3	3	4	4	4	4	4
	LD	4	4	4	4	4	4	4	4	4	4	4	4	4	4	4	4	4	4	4	4	4	4	4	4
M3	BF	4	4	4	4	4	4	4	4	3	1	1	1	1	1	1	1	1	2	2	4	4	4	4	4
	LD	4	4	4	4	4	4	4	4	4	4	4	4	4	4	4	4	4	4	4	4	4	4	4	4
S1	BF	3*	3*	3*	3*	3*	3*	3*	3*	1	1	1	1	1	1	0	0	0	0	0	0	0	0	0	3*
	LD	3	3	3	3	3	3	3	3	3	3	3	3	3	3	3	3	3	3	3	3	3	3	3	3
S2	BF	4	4	4	4	4	4	4	4	1	0	0	0	0	0	0	0	0	0	3	3	4	4	4	4
	LD	4	4	4	4	4	4	4	4	4	4	4	4	4	4	4	4	4	4	4	4	4	4	4	4
S3	BF	4	4	4	4	4	4	4	4	0	0	0	0	0	0	1	1	1	3	3	4	4	4	4	4
	LD	4	4	4	4	4	4	4	4	4	4	4	4	4	4	4	4	4	4	4	4	4	4	4	4

*Sometimes only 2 occupants slept at home.

Natural ventilation through windows is a useful mechanism for moderating indoor temperature and dispersing pollutants. Given the large number of influential factors such as wind direction, size, and location of openings, it is a complex engineering problem to analyse (Bazdidi-Tehrani et al. 2020; Chen et al. 2020). In the survey, occupants were asked to indicate the timetable and duration of the opening of windows in the dwellings. All participants reported opening windows once a day in winter and early spring and several times in summer. In winter, windows were mostly opened for just 10 to 20 min. In the summer, in contrast, windows were opened several times daily and between 4 and 10 h overnight. The ventilation time of the dwellings increased during lockdown, and all occupants reported that they opened the windows at 8:00 p.m., coinciding with the nightly lockdown clapping from balconies and windows (Tables 4 and 5).

**Table 4:** Window opening behaviour in summer and winter before and (after lockdown)

	Winter		Summer	
	Time frame (24-h system)	Duration (min)	Time frame (24-h system)	Duration (h)
M1	9 (9/20)	10 (20)	22–7	9
M2	8–9 (8–9/20)	20 (30)	22–7	9
M3	9–10 (9–10/20)	10 (30)	21–1	4
SI	9 (9/20)	20 (50)	21–5	8
S2	9 (9/20)	20 (40)	24–7	7
S3	8 (8/20)	15 (60)	21–7	10

**Table 5:** Window opening patterns before and after lockdown

Hour		1	2	3	4	5	6	7	8	9	10	11	12	13	14	15	16	17	18	19	20	21	22	23	24	Duration (min.)
M1	BF									X																10
	LD									X							X				X					60
M2	BF								X																	20
	LD										X										X					30
M3	BF									X																10
	LD									X											X					80
S1	BF									X																20
	LD									X						X	X	X			X					220
S2	BF									X																25
	LD											X	X	X							X					120
S3	BF								X																	15
	LD																X	X	X	X	X					180

For the questions pertaining to air conditioning systems, the survey results show that the use of cooling systems in Seville was sporadic, as these were only turned on occasionally to combat extreme temperatures. Most of the dwellings in Seville do not have heating systems, while all those monitored in Madrid have heating, some central, and the boilers were scheduled to operate from October to April. Occupants felt colder in winter in Seville, where central heating is generally lacking. Users in Seville also felt warmer in summer despite the presence of individual AC units in some rooms, not usually found in flats in Madrid.

### Thermal comfort and indoor air quality measurements

#### Temperature

Table 6 shows the mean values and standard deviations of air temperature for each season and dwelling are presented in Table 6. In winter, the mean living room temperature in Madrid was 17.4 °C (ranging from 14 to 19.5 °C) and less than 1 °C lower in the bedroom, with standard deviations ranging from ± 0.8 to ± 1.6 °C. In Seville, the mean living room temperature was 16.2 °C (ranging from 13 to 17.5 °C) and standard deviation ranging from ± 0.6 to ± 1.2 °C. Both the mean indoor temperatures and the standard deviations were lower in Seville than in Madrid in winter, despite the higher outdoor temperatures in Seville (4 to 6 °C higher on average) due to the less intense use of heating. In winter, the homes in Madrid with individual home heating systems (M1 and M2) and the 3 homes in Seville, one of which had an individual radiator system, were below 19 °C between 58 and 87% of the time.

**Table 6:** Statistics by case study, individual room of the dwelling and season (W — winter; Sp — spring; Su — summer; and A — autumn)

		Living room (day)				Bedroom (night)			
		W	Sp	Su	A	W	Sp	Su	A
M1	T [SD] (°C)	17.2 (1.6)	19.3 (2.3)	29.3 (1.5)	22.0 (3.0)	16.3 (2.4)	17.9 (1.1)	28.6 (1.4)	18.3 (2.3)
	RH [SD] (%)	56.9 (5.1)	50.2 (8.5)	33.0 (4.6)	54.6 (9.6)	61.9 (7.3)	57.0 (5.9)	35.8 (4.7)	67.4 (9.1)
	CO_2_ [SD] (ppm)	1425 (582)	859 (430)	454 (153)	862 (483)	1461 (582)	1139 (540)	466 (161)	1137 (591)
M2	T (SD] (°C)	17.6 (0.8)	19.9 (2.1)	26.7 (1.1)	21.2 (3.1)	17.1 (1.3)	19.9 (2.6)	27.7 (1.2)	20.5 (3.3)
	RH [SD] (%)	75.9 (4.3)	60.1 (13.3)	38.3 (5.4)	61.0 (14.3)	77.9 (6.8)	58.4 (14.3)	36.6 (4.7)	63.2 (15.3)
	CO_2_ [SD] (ppm)	2076 (706)	1157 (976)	439 (176)	1186 (1113)	2848 (1132)	1125 (1130)	445 (212)	1602 (1382)
M3	T (SD] (°C)	23.7 (1.1)	22.9 (1.9)	27 (1.7)	23.5 (1.9)	22.4 (1.0)	22.2 (2.3)	25.7 (1.8)	23.0 (1.5)
	RH [SD] (%)	36.8 (3.0)	38.4 (8.5)	33.7 (7.0)	39.4 (7.5)	40.3 (2.8)	42.0 (8.3)	29.0 (12.3)	41.0 (2.4)
	CO_2_ [SD] (ppm)	1034 (291)	804 (376)	446 (93)		1031 (290)	848 (381)	487 (90)	
S1	T (SD] (°C)	16.6 (0.8)	23.2 (3.1)	26.8 (1.4)	20.7 (3.1)	15.4 (1.1)	21.1 (3.1)	27.2 (1.5)	19.7 (3.3)
	RH [SD] (%)	64.9 (8.1)	53.6 (10.1)	49.8 (7.9)	67.0 (9.3)	59.3 (4.6)	58.4 (7.3)	50.8 (3.9)	64.7 (6.1)
	CO_2_ [SD] (ppm)	721 (285)	538 (175)	483 (86)	586 (212)	452 (88)	445 (232)	438 (35)	460 (187)
S2	T (SD] (°C)	15.1 (1.2)	22.3 (3.4)	29.4 (1.8)	23.2 (3.0)	14.5 (1.5)	22.3 (3.3)	27.1 (1.6)	22.9 (3.3)
	RH [SD] (%)	65.4 (6.7)	53.1 (10.6)	44.5 (9.1)	59.9 (9.1)	72.3 (6.3)	55.8 (10.2)	50.1 (8.2)	62.3 (9.1)
	CO_2_ [SD] (ppm)	733 (503)	521 (298)	444 (77)	523 (324)	1182.3 (461)	774.1 (1325)	596 (576)	604 (848)
S3	T (SD] (°C)	17.0 (1.2)	25.5 (1.8)	27.8 (1.9)	25.5 (1.0)	16.4 (1.1)	24.2 (4.0)	28.5 (2.0)	24.7 (1.7)
	RH [SD] (%)	65.0 (6.1)	51.9 (7.4)	47.8 (6.8)	60.1 (4.2)	67.6 (5.5)	55.8 (11.5)	46.2 (7.3)	61.0 (7.6)
	CO_2_ [SD] (ppm)	402 (523)	680 (352)	462 (197)	691 (266)	1059 (523)	753 (463)	465 (197)	690 (352)

In summer, the mean living room (LR) temperature in Madrid and Seville was approximately 28 °C (ranging from 26.5 to 30 °C), while the bedroom (B) temperature in Seville was around 0.5 °C lower. In both cases, the mean standard deviation was slightly higher (+ 0.2 °C) in Seville, mainly due to the greater daily thermal oscillation in summer in Seville. Although in summer the outdoor temperatures are usually higher (4 to 5 °C on average) than in Madrid, both cities displayed similar values in the year of this research. In summer, these dwellings are above 26 °C for 32% and 71% of the time. The results for both winter and summer reveal conditions of energy poverty, housing precariousness, and, in general, social thermal vulnerability in indoor spaces.

**Fig. 3:**
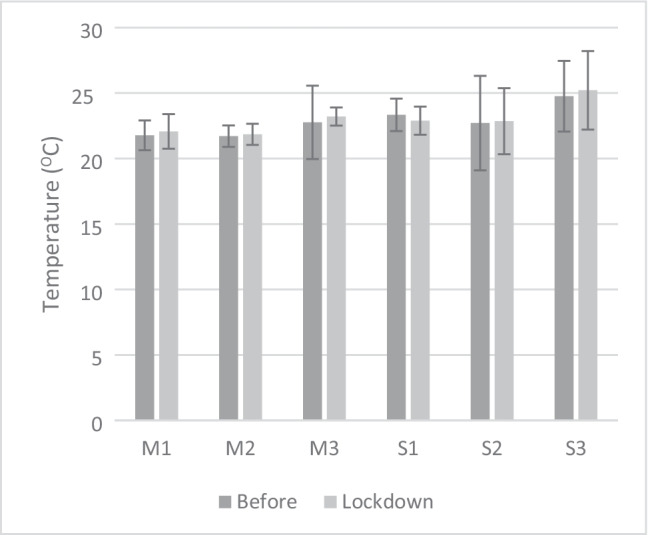
Mean temperatures of dwellings in Madrid (M) and Seville (S) before (dark grey) and after lockdown (light grey)

In spring and autumn, indoor temperatures were closer to the thermal comfort zones for seated occupants with common clothing insulation, according to EN-ISO 7730 7730 (CEN—European Committee for Standardization 2005): around 19.8 to 22 °C in Madrid and 22 to 24 °C in Seville (Fig. 3), with a higher mean standard deviation in the case of Seville (ranging from + 1.8 to + 4.0 °C in Seville regards to + 1.5 to + 3.3 °C in Madrid).

During the pandemic, a distinction should be established between the different states of the dwellings in Madrid and Seville. In Madrid the heating operated until April 10 in these dwellings, while in Seville, dwellings did not have heating systems in operation. Furthermore, outdoor temperatures were higher in Seville during this period.

There is no difference in outside temperature for the same period before and during lockdown in Seville and Madrid. In Madrid, the average indoor temperature before and after lockdown varied, but not enough to be considered statistically different (*p*-value > 0.05). This can be attributed to the use of heating systems, as the lockdown period was a little cooler. The daily average temperature oscillation was 14 °C. The average interior temperature of the dwellings varied between 23 and 26° C, the lowest average temperature of the dwellings during the lockdown period. This can be attributed more to external variation than to user-specific behaviour changes. In Seville, the data for before and after lockdown are similar (Fig. 3).

### CO_2_ concentration

The maximum recommended carbon dioxide threshold of 1200 ppm (CEN—European Committee for Standardization 2017) was exceeded in all households during the winter and summer measurement periods, as seen in Table 7. Values of over 1200 ppm were found 42 to 84% of the time in all dwellings except S1. Values of > 2000 ppm were recorded in the same households but most significantly in M2 (23% of winter hours with peaks as high as 4000 ppm) and S2 (37% of winter hours and peaks of up to 5000 ppm). In summer, values of over 1200 ppm were observed around 6% of the time except in dwellings M2 and S2, where the percentage logged was much higher, as in winter. In S2 this could be explained by summertime ventilation practice and in M2 by high airtightness levels.

**Table 7:** Percentage of hours that CO_2_ concentration is within a range in winter and summer

	% (400–600 ppm)	% (600–1200 ppm)	% (1200–1600 ppm)	% (1600–2000 ppm)	% (> 2000 ppm)
W-M1	6.3	29.0	29.9	17.4	17.4
W-M2	5.8	10.1	56.5	4.5	23.0
W-M3	14.0	43.9	24.1	15.9	1.9
W-S1	23.2	71.0	5.8	0.0	0.0
W-S2	20.8	31.4	6.3	4.5	37.0
W-S3	12.6	33.8	24.6	20.3	8.7
S-M1	79.0	17.8	3.2	0.0	0.0
S-M2	50.2	21.5	4.1	5.2	19.0
S-M3	59.4	34.7	0.0	5.0	0.9
S-S1	93.1	0.0	6.8	0.0	0.0
S-S2	42.5	18.7	4.1	24.2	10.5
S-S3	63.5	29.7	4.6	0.9	1.4

**Fig. 4:**
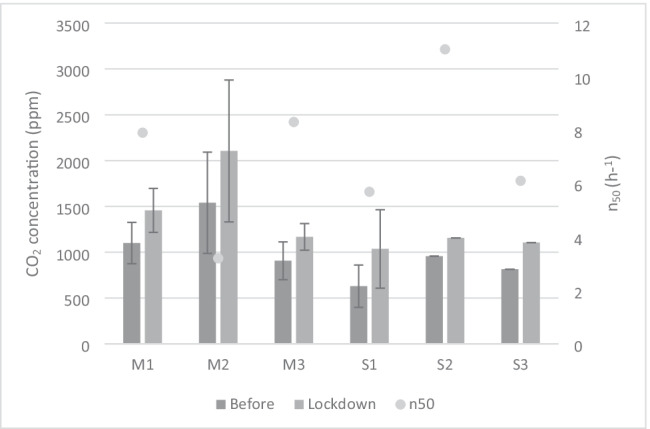
Mean value of CO_2_ concentration of dwellings in Madrid (M) and Seville (S) before and after lockdown

During lockdown, all dwellings reported higher levels of CO_2_ concentration, as the occupants stayed at home throughout the day. In case studies M1, M2, and M3 the indoor CO_2_ concentration increased by 32%, 37%, and 28% respectively, with the most airtight dwelling showing the highest increase. In Seville, in case studies S2 and S3, the indoor CO_2_ concentration increased by 20% and 35% respectively. The results were especially significant in S1 where concentration was 64% higher than the average value during lockdown (Table 8; Fig. 4).

**Table 8:** Percentage of hours that CO_2_ concentration is within a range before and during lockdown

	% (400–600 ppm)	% (600–1200 ppm)	% (1200–1600 ppm)	% (1600–2000 ppm)	% (> 2000 ppm)
M1-Before	41.6	24.4	16.5	8.4	9.1
M1-Lockdown	12.6	21.5	41.1	12.1	12.7
M2-Before	29.0	9.7	36.0	4.3	21.0
M2-Lockdown	0.0	19.0	46	6.4	28.7
M3-Before	39.1	37.0	12.0	10.5	1.4
M3-Lockdown	20.1	31.0	28.9	12.3	7.7
S1-Before	58.2	35.5	6.3	0.0	0.0
S1-Lockdown	38.4	18.2	26.5	15.3	1.6
S2-Before	31.6	25.1	5.2	14.4	23.7
S2-Lockdown	16.0	12.5	25.8	18.6	27.1
S3-Before	38.0	31.7	14.6	10.6	5.0
S3-Lockdown	12.3	38.0	29.3	12.2	8.2

Due to the COVID-19 emergency, different groups of experts have set a more restrictive limit of 800 ppm for CO_2_ concentration indoors with non-cohabitants (J. Allen et al. 2020; Ministerio de Sanidad del Gobierno de España 2020; Ruiz de Adana et al. 2021). As this protocol is based on the fact that those possibly infected can transmit the disease asymptomatically, it seems reasonable to establish a more demanding CO_2_ concentration limit during the pandemic in spaces shared with cohabitants.

### Surveys

#### Mental health

From the start of the pandemic until today, no sample participant has been treated by a psychologist or psychiatrist due to any psychological problems. None have reported chronic (long-term) illnesses unrelated to COVID-19 or any instances of anxiety or panic attacks.

Two people reported that thoughts, memories, or images of coronavirus have altered their social life or relationships with family or friends. Only one person reported feeling more depressed during lockdown and worried about their professional future at work.

In case study M3, a close relative was hospitalized for COVID-19, and in case study S2, two close relatives were asymptomatic, but none lived in the homes studied. Everyone knows people who have died from COVID-19, particularly the elderly.

#### Occupant perception

In Madrid, the temperatures in bedrooms and living rooms were perceived to be close to comfort levels in spring, autumn, and winter. In Seville, however, both winter and summer levels lay 1.3 points outside the comfort range, with users reporting discomfort, particularly at night. Indoor draughts were nearly imperceptible, even in the homes with cross ventilation. In Madrid, indoor odours were negligible, whereas Seville residents reported odours inside dwellings, particularly in winter when windows were closed for most of the day, as shown on Table 9.

**Table 9:** Summary of occupant perception (W = winter, Sp = spring, Su = summer, and A = Autumn)

	Madrid				Seville			
	W	Su	A	Sp	W	Su	A	Sp
Bedroom temperature (1–5) sleep	2.8	4.0	2.7	2.0	1.7	4.3	3.3	3.3
Living room temperature (1–5)	3.0	3.5	2.7	3.0	2.3	3.8	3.0	3.0
Draught in bedroom (1–5)	1.8	2.5	1.3	1.3	1.5	1.3	1.3	1.5
Draught in living room (1–5)	1.8	1.8	1.0	1.0	1.5	1.3	1.3	1.5
Odour in bedroom (1–5)	1.0	1.0	1.0	1.0	2.8	1.3	2.0	2.0
Odour in living room (1–5)	1.0	1.0	1.0	1.0	2.3	1.8	1.5	1.5
Symptoms	Men: nasal congestion, slight headache	Men: dry mouth, fitful sleep, tiredness the following day				Women and men: Headache		
		Women: dry mouth, fitful sleep, tiredness the following day						
Sleep quality	Good	Average	Good	Good	Good	Average	Good	Good

Most occupants were unable to distinguish when the air was stuffy and expressed surprise at the CO_2_ readings recorded by the dataloggers. More discomfort related to high CO_2_ concentrations was reported by the occupants of the most airtight flat (M2). The symptoms described by the female occupants of that flat included fitful sleep and tiredness the following day. The male occupants reported nasal congestion and a slight headache, and in summer a dry mouth, fitful sleep and tiredness the following day. In Seville, symptoms were only reported for summer nights.

**Fig. 5:**
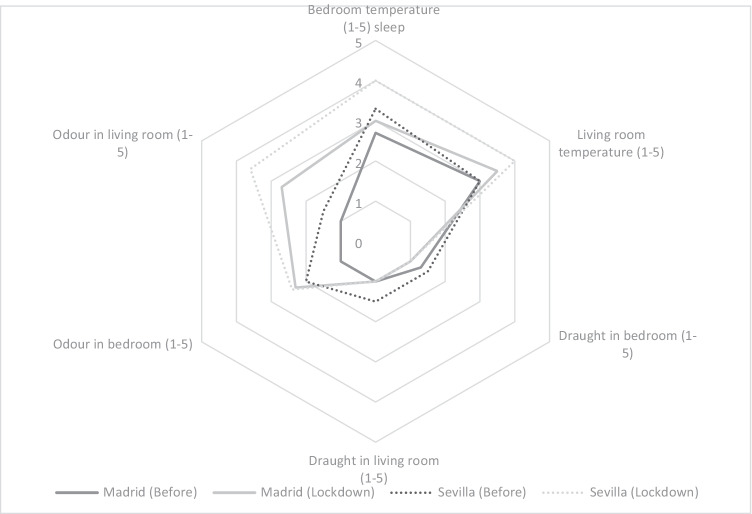
Average perception of occupants in spring before and during lockdown

A comparison was carried out for the results of the surveys for the same period in 2019 and the results of the surveys during lockdown in 2020. The score given to the different indicators on comfort increased during lockdown in general terms (Fig. 5).

With respect to the temperature of the bedroom during the night and the living room during the day, the sensation of heat increased in all the case studies but more so in Seville. Air draught sensation is the only parameter that decreased very slightly. However, the perceptions of odour inside the home increased by almost 2 points, in both the daytime and nighttime areas. This was mainly due to the fact that more cooking was done in the dwellings, although occupants also became more sensitive to odours during lockdown, perhaps searching for distraction or the feeling that they were closed in, which was not the case previously.

**Fig. 6:**
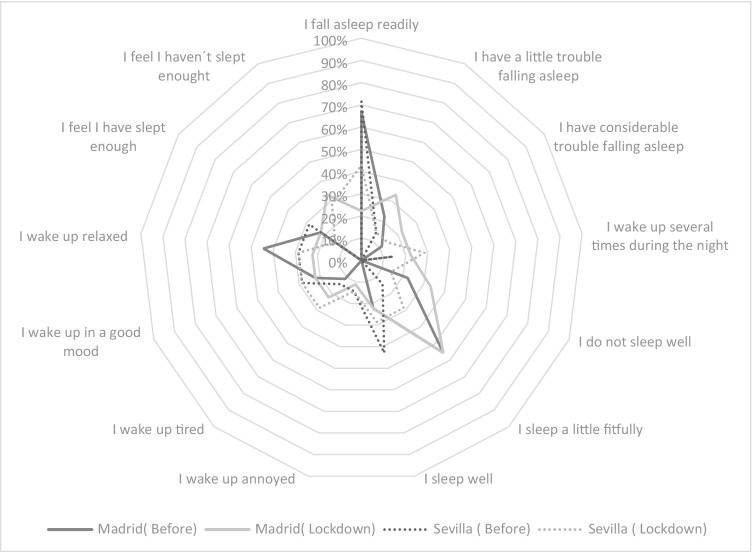
Effect on occupants’ sleeping pattern in spring before and during lockdown

An increase in sleep quality impairment was identified during lockdown by occupants who found it harder to fall asleep, were restless while sleeping and got up several times, sometimes even tired, and upset. They gave the main reasons as stressful situations (working from home, caring for children and the elderly, increased number of household chores and cooking) and uncertainty (concern about not knowing what was happening and how events would unfold) that they felt during lockdown (Fig. 6).

In spring of the previous year, no symptoms were reported (Table 9). However, during lockdown in Madrid, occupants reported a series of symptoms such as dryness in the nose, mouth, skin, and eyes and thirst. In Seville, the symptoms were nasal congestion, thirst, and watery eyes.

## Discussion of the findings

### Ventilation, air temperature and CO_2_ concentration

Typical thermal perception in bedrooms and living rooms of dwellings of Madrid was usually perceived close to comfort level in both winter and mid-seasons. However, in Seville, mean air temperature values were usually below 17 °C in winter and around 28 °C during summer so that inhabitants routinely reported discomfort, particularly at night. In contrast, during the lockdown period, the heat perception increased in all case studies, but was more pronounced in Seville, probably due to the continuous occupation of the home and the increase in internal loads. The time spent at home and number of simultaneous inhabitants increased significantly during the lockdown period.

Several studies have found that the body’s thermoregulatory system is closely related to sleep quality, and dysregulation of thermal comfort can therefore impair sleep quality [69]. Warm feet reduce the amount of time it takes to fall asleep (Kräuchi et al. 1999). In hot environments, cooling the back and neck improves both sleep quality and efficiency (Lan et al. 2018). Thermal discomfort can affect total sleep time, sleep state maintenance, REM cycle length, and sleep efficiency (Lan et al. 2017). During health emergencies such as COVID19, this aspect becomes highly significant due to the strong correlation between sleep quality/disorders, predisposition to infection and disease progression. Thus, optimizing thermal environment to improve thermal comfort and perception is crucial to enhancing sleep quality and related health conditions.

CO_2_ concentration is one of the IAQ parameters that play a key role in inhabitants’ health and wellbeing (CO_2_) (Altomonte et al. 2020). The primary sources of CO_2_ inside dwellings are the occupants. Levels above approximately 950 ppm begin to impair cognitive performance by 15%. At levels above 1400 ppm, cognitive performance is reduced by 50%, and fatigue and poor judgement also set in Altomonte et al. (2020), while lethal levels of 250,000 ppm can cause death [41] (although these are very rare inside homes except in cases of accident). Frequent fresh air exchange, opening windows and doors, or operating ventilation systems results in a dilution of CO_2_ preventing it from accumulating through the entry of outdoor air.

In winter, in the cases under study, CO_2_ values were above the recommended threshold of 1200 ppm most of the time. Windows were mostly opened for just 10 to 20 min per day during the cold season, and overall ventilation mainly relied on air leakage through the envelope. However, in summer, on average, CO_2_ concentration was above this figure just 6% of the time. During the warm season, windows were opened several times a day, often remaining open for most of the day, and on average were open between 4 and 10 h overnight. In any case, merely opening the windows does not ensure proper ventilation, as this is conditioned by the weather.

On average, overall IAQ before lockdown was poor compared to the European situation but worsened to some extent during lockdown.

Winter mean values for indoor air temperature are close to the limit of thermal stress due to cold. Often, especially in Seville, dwellings lack suitable heating facilities and mostly rely on portable heaters with high energy consumption. This determines a strong relation between poor ventilation and energy conservation in the dwellings, an effect which is more pronounced in the bedrooms at night. In contrast, the higher ventilation rate observed in summer helps to dissipate overheating both at night-time and in the early morning.

During lockdown, all dwellings report higher levels of CO_2_ concentration than previously under typical operation, with figures of indoor CO_2_ concentration increasing by more than 30%. Although during the lockdown period windows were opened more often, the continuous presence of the inhabitants in the home throughout the day also contributed to increasing these values.

## Mental health and IAQ occupant perception

In the prolonged stay-at-home period, most occupants were unable to discern when the air was stuffy, mainly due to olfactory fatigue (Kadohisa and Wilson 2006), as they lacked the ability to identify inappropriate atmospheres or to perceive when these needed to be renewed.

This could also be linked to the halo effects described at home. This cognitive error in judgement reflects one’s individual prejudices, ideology, and social perception. Before lockdown, people perceived their home as an idealized safe place where they could be safe from pollution and the outside elements. People refer to the tendency to assign positive qualities like clean and pure air indoors with no specific evidence or real assessment. These findings often appear in different studies focused on residents and their exposure to and perception of air pollution risks in their homes. However, further research is required with studies exploring the ability of participants to perceive indoor air quality, combining perceptions of air quality with objective measurements of IAQ. Residents consistently overestimate air quality in their homes (Boso et al. 2020; Hofflinger et al. 2019). During lockdown, the production of odours and internal contaminants increased, as did adaptation and numbed perception. However, the increased time spent at home also increased emission sources and activity. More frequent cooking was responsible for many odours and pollutants.

This situation led to an increase in symptoms and worsened the well-being of the inhabitants. Some of the symptoms described by users (headaches, fitful sleep, and tiredness) could be related to heightened levels of anxiety due to the pandemic, accompanied by higher CO_2_ concentrations and seasonal allergies (especially during spring).

In this study, during the lockdown period, occupants reported increased sleep disorders, mostly attributable to stressful situations and uncertainty (Antunes et al. 2020; Florea et al. 2021; Meaklim et al. 2021; O’regan et al. 2021; Pérez-Carbonell et al. 2020; Scarpelli et al. 2021). Furthermore, there was a deterioration of indoor ambient conditions, not only IAQ but also other comfort-related parameters. Several studies show that sleep disturbances have affected a large proportion of the general population during the COVID-19 pandemic lockdown. These results are correlated with a self-assessed impact on mental health but may also be associated with suspected COVID-19 infection, changes in habits, and self-isolation (Pérez-Carbonell et al. 2020). Additionally poorer sleep quality during lockdown has been linked to a decrease in sunlight exposure, lack of daily exercise, and increased smartphone usage before bedtime. In fact, poorer sleep quality and higher levels of loneliness have been found to affect the relationship between stress and mental health symptoms (depression, anxiety), highlighting the critical, but modifiable, impact of sleep on mental health during lockdown (Varma et al. 2021).

Meaklim et al. (2021) did a cross-sectional survey completed by 2724 participants from 67 countries during the COVID-19 pandemic. The results showed that post-pandemic insomnia symptoms were associated with higher levels of stress, anxiety, and depression than pre-existing or no insomnia symptoms. Across all groups, individuals reporting a previous mental health diagnosis had worse stress, anxiety, and depression than those without a previous mental health diagnosis (Meaklim et al. 2021).

Therefore, the population, who took refuge in their homes due to the uncertainty of the health situation, complying with government regulations, began to perceive their homes as a space that is no longer so safe. This situation raised interest in aspects of home IAQ which had previously been unnoticed by inhabitants.

Although the ability of homes to provide shelter was initially assumed, the confusion and lack of accurate knowledge on COVID transmission introduced an additional anxiety component to the perception of homes. This aspect evolved from the beginning of lockdown with a significant impact on the IAQ management of the dwellings ranging from fear to introduce outdoor air, fuelled by the government’s decision to spray disinfectants on the streets, to an overuse of home disinfectants and chemicals, and more outlandish practices such as ozone liberation.

These aspects highlighted growing concern among inhabitants on the actual environmental safety of their homes, also in relation to the dilemma of energy conservation.

One of the most significant aspects was the users’ discovery that most homes were unable to provide healthy and safe environments. This led to an increased awareness of the need for environmental quality control tools, since individual perception and manual control of the indoor environment was shown to be very limited in achieving these requirements, judging by the interviews with the occupants.

## Conclusions

Many countries, including Spain, imposed lockdown as a means of controlling the spread of COVID-19. As a result, people spent most if not all of their time during lockdown inside their homes. This study evaluated the influence of the occupants’ behaviour on indoor thermal comfort and air quality and their perception of it before and during the lockdown period. Environmental variables, including air temperature, relative humidity, and CO_2_ levels, were monitored in six representative dwellings to identify the times in which these variables were outside the range for healthy values. The results revealed a lack of ventilation in the dwellings studied, especially during winter, when ventilation depends mainly on air leakage through the envelope, given the scant ventilation reported by users. Of particular interest were the findings for the most airtight flats in Madrid, where values of over 2000 ppm were recorded 23% of the time. In Seville, the highest values were observed in a dwelling with medium airtightness in which occupants reported that they closed the bedroom door at night. These higher CO_2_ concentrations can also be correlated to a reduction in concentration on task development, as well as an increased risk of airborne disease transmission.

The study focuses on low-income housing with limited access to high-end HVAC systems or even to mechanical or controlled ventilation. These dwellings are near the fuel poverty threshold, so energy conservation is a key aspect of home management. Home IAQ was not initially perceived as an issue by inhabitants but awareness has awakened due to lockdown.

Before lockdown, the occupants were unable to perceive the changes in indoor CO_2_ concentration and expressed surprise at datalogger readings. However, they were keenly aware that in the absence of mechanical ventilation systems, they needed to open windows to ventilate their flats, especially early in the morning. However, the typical routine was only to open these for a short time, 10 to 20 min. Continuous distributed ventilation was not considered necessary for IAQ control.

During lockdown, occupants progressively became more sensitive to IAQ as it was something that they thought about and worried about during this period. More time was spent opening windows and in contact with the air outside. There was also an increase in symptoms in the spring, when headaches, thirst, and disturbed sleep were more frequent. Initially, these symptoms cannot be differentiated from those caused by the change in IAQ and anxiety over the present COVID-19 pandemic.

An increase was noted in sleep disorders among occupants, mostly attributed to stressful situations (working from home, caring for children and the elderly, more housework and cooking) and the uncertainty of not knowing what was happening and how things would turn out during lockdown. These findings and the current COVID-19 pandemic have led us to reconsider the living space in dwellings, which should incorporate more uses, especially for working from home given the social distancing imposed.

Anticipating a scenario where greater use is made of the dwelling the following findings are being developed:

- Based on the analysed cases, inhabitants need to increase users’ awareness of the importance of maintaining acceptable minimum levels of natural ventilation required for a healthy indoor environment and indoor comfort.
- The widespread use of measurement devices is necessary, and CO_2_ meters are the minimum equipment for indoor control in homes. Personal perception has shown users’ inability to provide safe and accurate control even at home.
- At present, the existing housing stock urgently needs to be retrofitted, not only to provide better energy-conservation capabilities, but also to include suitable ventilation systems which both guarantee air changes indoors and filter air coming in from outside. Self-confidence and resilience at home must be boosted to provide the population with enough shelter capacity in the event of emergencies and health crises.
- Methods other than airtightness should be considered to achieve indoor thermal comfort. These methods should be suitable and affordable for people on low incomes.

## Data Availability

The datasets generated during and/or analysed during the current study are available in the ONEDRIVE repository of the University of Seville: https://uses0-my.sharepoint.com/:b:/g/personal/jfernandezaguera_us_es/EegcHtaU4pZHgihjsL-FswkBwyRoZ8USHmcsaG_osmir_g?e=jNNtMr

## Appendix

Surveys
